# Seed Dispersal as a Multiphase Process: Integrating Abiotic and Biotic Vectors Across Ecological Gradients

**DOI:** 10.1002/ece3.72564

**Published:** 2025-11-27

**Authors:** Fabián Alejandro Rubalcava‐Castillo, Martha Susana Zuloaga‐Aguilar, Luis Ignacio Íñiguez‐Dávalos, Víctor Manuel Martínez‐Calderón, Joaquín Sosa‐Ramírez

**Affiliations:** ^1^ Departamento de Ecología y Recursos Naturales, Centro Universitario de la Costa Sur Universidad de Guadalajara Autlán de Navarro Jalisco Mexico; ^2^ Dirección Académica de Negocios y Agricultura Universidad Tecnológica del Norte de Aguascalientes Aguascalientes Mexico; ^3^ Centro de Ciencias Agropecuarias Universidad Autónoma de Aguascalientes Aguascalientes Mexico

**Keywords:** diaspores, diploendozoochory, dispersal systems, endozoochory, multiphase seed dispersal

## Abstract

Seed dispersal is a dynamic process through which diaspores (seeds or seed‐bearing fruits) are detached from the mother plant, transported to different sites in the landscape that offer physical protection, competitive advantages, or lower predation risk. A variety of biotic and abiotic factors contribute to seed dispersal processes, resulting in a high diversity of dispersal systems observed in nature. At present, the relationship and classification of seed dispersal processes remain unclear. It is therefore essential to delineate seed dispersal systems and understand their functional traits in relation to ecosystem functioning and diversity, in order to elucidate plant distribution patterns. This review presents an updated synthesis of current knowledge on multiphase seed, reframing diaspora dispersal systems (fruits with seeds) as dynamic networks of transitions rather than discrete events, with particular emphasis on their efficiency and legitimacy. Three operational phases were defined: primary release, secondary transit and deposition, and the filters acting at each stage, including physical abrasion, digestive modification, vector movement, and habitat boundaries. To achieve this purpose, an analysis of 115 bibliographical references was conducted, ensuring the inclusion of seminal works from preceding years. We specifically describe how endozoochory and diploendozoochory can be considered efficient and legitimate dispersal systems for plants, as well as the potential benefits of a triple endozoochory process. Likewise, we propose a network‐based framework to model multiphase dispersal, integrating movement ecology, gut retention, and seed condition metrics. This review demonstrates that diaspore dispersal is a multifactorial process associated with intrinsic attributes of the diaspores, their dispersion agents, and their interactions with the environment and proposes a subtype of seed dispersal by endozoochory (triploendozoochory). By highlighting the ecological relevance and conservation implications of multiphase dispersal, this review calls for interdisciplinary research to quantify its contribution to plant connectivity, especially under global change. Recognizing predators, waterbirds, and caching agents' key multiphase vectors reframes their role in ecosystem resilience and restoration.

## Graphical abstract

Raccoon specimen, seed disperser through endozoochory.

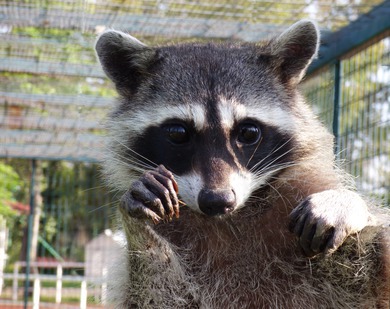

## 1 Introduction

### 1.1 Seed Dispersal and Plant Migration

Seed dispersal or translocation of diaspores is a dynamic transport process operating across multiple scales. It involves diverse biotic and abiotic agents, facilitating the movement of seeds away from the parent plant to sites that offer physical protection, competitive advantages, or lower predation risk (Beckman and Sullivan 2023; see “Directed dispersal hypothesis” by Howe and Smallwood 1982; Noir et al. 2002). According to Howe and Smallwood (1982) and Traveset et al. (2014), dispersal of diaspores contributes to the achievement of three fundamental ecological objectives: first, to avoid competition between seedlings and the mother plant for limiting resources; second, to prevent intra‐specific density‐dependent competition (competition for the same resources); and third, to decrease the likelihood of predation due to density dependence near the mother plant. The hypothesis posits that seedlings established farther from the mother plants will have a greater likelihood of evading predation and surviving intra‐specific competition, known as the “escape hypothesis” (Howe and Smallwood 1982). Understanding dispersal patterns can help elucidate the spatial dynamics of plant recruitment, the mechanisms of forest ecosystems' regeneration (Howe and Smallwood 1982; Levin et al. 2003), and the colonization of new areas within regional distributions. In plant ecology, recruitment refers to the successful establishment of new individuals into a population in a landscape, typically through seed germination, seedling survival, and growth until they reach a defined life stage (Eriksson and Ehrlén 2008; Alcántara et al. 2025). This process is critical for maintaining population stability and shaping community composition, and it reflects the transition from dispersal to demographic integration. Similarly, the dispersal pattern of diaspores can effectively reflect the geographic distribution of species (McGill et al. 2006; Westoby and Wright 2006); recent evidence suggests a combination of non‐random association and significant stochasticity in seed dispersal processes (Costa et al. 2014; Green et al. 2022; Heleno and Vargas 2015; Higgins et al. 2003; Vargas et al. 2023).

Diaspores are defined as the plant parts that are dispersed, including seeds, fruits, infructescence, and other dispersal units (Beckman and Sullivan 2023; Zhu and Liu 2012). Therefore, what constitutes a diaspore depends on the plant species and its dispersal mode. Diaspores are propagules that detach from the parent plant and reach the substrate; they may subsequently disperse through multiple morphological adaptations that enhance their dispersal capacity (Mamut et al. 2023; Qu et al. 2024). The dispersal pattern of diaspores is directly associated with the dispersal system (Rumeu‐Ruiz et al. 2014). A dispersal system is a fundamental ecological process that enables the translocation of plant diaspores from the parent plant to spread and colonize new areas. Accordingly, dispersal systems function as abiotic and biotic environmental filters, shaping the spatial patterns of plant population establishment (Nathan and Muller‐Landau 2000), the movement of genetic material through gene flow, and the resulting population genetic structure (Niembro 1982; Bacles et al. 2006; García and Grivet 2011).

The phenomenon of seed dispersal, a critical ecological process, is mediated by a variety of mechanisms, one of the more remarkable being endozoochory, wherein plant species attract diverse frugivore assemblages. This interaction generates intricate dispersal patterns, shaped by the unique behavioral, morphological, and physiological traits of the frugivores involved (Jordano and Schupp 2000; Schupp et al. 2010). A thorough understanding of diaspore dispersal mechanisms is paramount for the development of robust management and conservation strategies at population, community, and ecosystem levels.

While acknowledging the existence of a spectrum of legitimate dispersal events (Garrote et al. 2025; Heleno et al. 2011; Montesinos‐Navarro et al. 2017), we propose a dichotomous classification for individual disperser species or functional groups of legitimate dispersers. This simplification, despite recognizing the inherent continuum, offers a more tractable framework for elucidating relationships and functional distinctions among diverse seed dispersal systems.

The present review aims to synthesize the extant literature on the factors influencing seed dispersal systems, with reference to the role of the intrinsic attributes of diaspores, the characteristics of dispersers, and their environmental interactions. The ultimate objective of this synthesis is to demonstrate the efficacy and ecological significance of these dispersal systems in shaping ecosystem functioning and biodiversity.

## 2 Materials and Methods

A review was conducted to synthesize the current knowledge on seed dispersal systems. A structured, replicable, and comprehensive approach was chosen, employing a multi‐stage process that included the identification, selection, and eligibility assessment of studies. By minimizing bias through meticulous literature searches, this approach ensures transparency and scientific rigor while providing a detailed account of the research procedures. This review specifically focuses on papers that analyze diverse seed dispersal systems, aiming to deliver a contemporary and comprehensive overview of recent advancements in this evolving field.

The research question for this study was structured using a modified PICO framework, on the basis of the methodology proposed by Nishikawa‐Pacher (2022). This approach employs an acronym to represent the problem (P), the intervention (I), the comparison (C), and the outcome (O). Specifically, in our work, “P” represents the need to understand dispersal systems to demonstrate their efficacy and legitimacy in ecosystem regeneration; “I” designates the demonstration that diaspore dispersal is a multifactorial process, intricately linked to the intrinsic attributes of diaspores, dispersers' characteristics, and environmental interactions; “C” entails the comparative delineation of the seed dispersal systems and the analysis of their functional traits in relation to ecosystem functioning and biodiversity; finally, “O” aims to synthesize the knowledge of primary and secondary diaspore dispersal systems (fruiting units) demonstrating their effectiveness and legitimacy in ecological regeneration processes.

Accordingly, the research question guiding this study is: How do the intrinsic attributes of diaspores, the characteristics of dispersers, and their interactions with the environment shape each seed dispersal system, thereby determining its effectiveness and contribution to ecosystem functioning and biodiversity?

### 2.1 Search Strategy and Databases

A comprehensive literature search was executed across the electronic databases Web of Science, Scopus, SpringerLink (Solar), and Google Scholar between January 2024 and June 2025 to identify relevant articles for this review (Table 1). Inclusion criteria were rigorously defined to ensure the selection of high‐quality and pertinent studies of the indexed journals. We used the following keywords and Boolean connectors:

**TABLE 1:** Summary of initial retrieval of articles by database.

Database	Search string summary	Records retrieved
Web of Science	(seed dispersal OR barochory… OR polychory) AND (diaspore OR seed)	1245
Scopus	(seed dispersal OR barochory… OR polychory) AND (diaspore OR seed)	989
SpringerLink (Solar)	(seed dispersal OR barochory… OR polychory) AND (diaspore OR seed)	462
Google scholar	(seed dispersal OR barochory… OR polychory) AND (diaspore OR seed)	1520

Seed dispersal OR barochory OR anemochory OR hydrochory OR autochory OR allochory OR zoochory OR entomochory OR myrmechochory OR endozoochory OR diploendozoochory OR epizoochory OR synzoochory OR polychory. Each database query combined these terms with “AND (diaspore OR seed)” to focus on dispersal units. Duplicates were removed prior to screening.

### 2.2 Screening and Selection

Duplicates removed: 862

Title/abstract screening: 2354 → 412

Full‐text assessment: 412 → 115 included

Eligible articles encompassed original research, review articles, and scholarly popularizations. Furthermore, only articles with titles and abstracts demonstrably relevant to the study's focus on seed dispersal were included. Conference proceedings, letters, errata, notes, and studies without explicit dispersal data were excluded.

### 2.3 Justification for Seminal Literature

Although the review prioritized the period 2015–2025, earlier works were incorporated to provide historical context and clarify key concepts that remain relevant today. This ensures conceptual continuity and acknowledges pioneering contributions.

### 2.4 Final Reference Corpus

The review integrates 184 bibliographical references. Each citation was classified as “recent” (2015–2025; *n* = 50) or “seminal” (pre‐2015; *n* = 65), aligning with the total reported in the manuscript. No supplementary files were needed, as all included studies are explicitly listed in the reference section.

## 3 Dispersion by Abiotic and Biotic Agents

**FIGURE 1:**
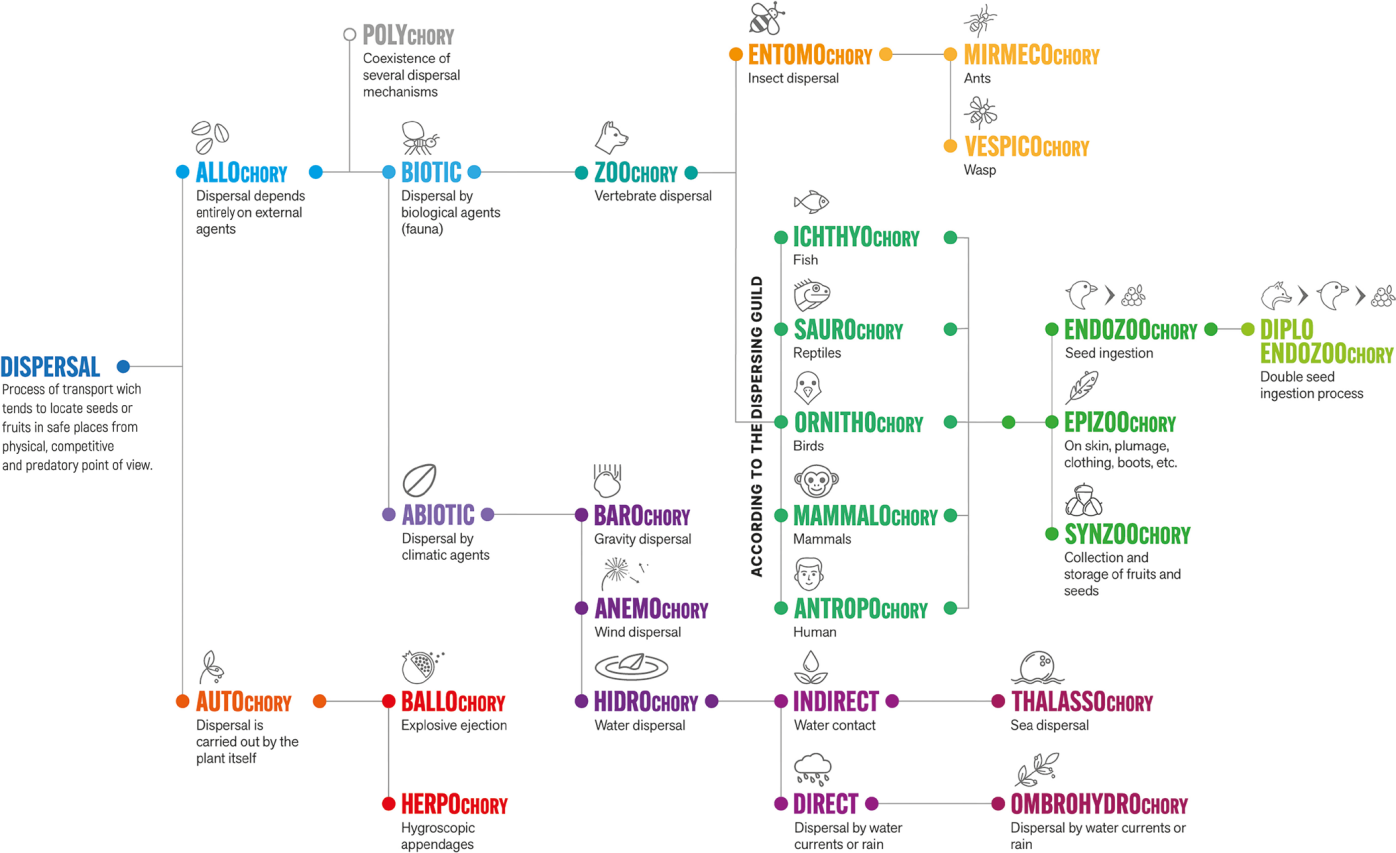
Conceptual map illustrating the dispersal systems, classified according to their principal driving forces.

Abiotic dispersal can be broadly classified into three main categories: barochory, anemochory, and hydrochory (Figure 1). Of these, hydrochory can be further subdivided into two subtypes: ombrohydrochory and nautohydrochory.

### 3.1 Dispersion Through the Force of Gravity: The Barochory

Barochory is the passive fall of diaspores under gravity; it typically involves relatively large, simple seeds that drop and often roll or bounce near the parent plant (Zona 2017; Traveset et al. 2014). Barochorous dispersal usually produces short‐distance seed shadows and high local density, increasing intraspecific competition and post‐dispersal predation risk; nevertheless, many barochores are subject to secondary phases (e.g., removal by rodents) that extend dispersal distance and influence recruitment (Calva‐Soto et al. 2022; Luo et al. 2023; Puerta 2008; Ramos et al. 2017; Rubio‐Licona et al. 2011; Vittoz and Engler 2007).

### 3.2 The Movement of Seeds by Wind: The Anemochory

Anemochory is wind‐mediated dispersal relying on morphological specializations (wings, pappus, hairs) that increase lofting and ride updrafts or horizontal winds (der Weduwen and Ruxton 2019; Nathan et al. 2008; Tackenberg et al. 2003). Wind‐driven dispersal can produce long‐distance events when atmospheric conditions and seed traits align, but outcomes are strongly modulated by habitat heterogeneity, surface roughness, and episodic weather; when wind is insufficient, seeds with mixed traits may resort to animal‐mediated secondary dispersal (Abid et al. 2024; Seale and Nakayama 2020; Qu et al. 2024).

### 3.3 The Continental Water Bodies as a Dispersion Medium: The Hydrochory

The term “hydrochory” is the dispersal of seeds by fresh water (Figure 1) (van Rheede van Oudtshoorn and van Rooyen 1999; Zona 2017). This form of dispersal can be considered long‐distance when the seed has specializations and structures that allow it to float (air chambers, spongy tissue, impermeable cuticles) and be displaced by the current for a longer period (nautohydrochory) or mobilized locally by rain (ombrohydrochory) (Seiwa et al. 2008; van Rheede van Oudtshoorn and van Rooyen 1999; Nathan et al. 2008; Sánchez‐Salas et al. 2017; Romero‐Méndez et al. 2018). Hydrochorus dispersal can act as a primary or secondary phase (e.g., endozoochory plus hydrochory), but digestion often reduces buoyancy or viability, so sequential interactions can alter hydrochory effectiveness (Navarro‐Ramos et al. 2024; Romero‐Méndez et al. 2018).

### 3.4 Two Strategies of Seed Dispersal Beyond Abiotic Factors

The success of seed dispersal in a plant is mainly attributed to two primary strategies: (1) whether the plant can disperse its own seeds without assistance, or (2) whether the dispersal of the plant depends entirely on external agents. As Biswas and Bordolui (2021) accurately state, this distinction leads to two general types of seed dispersal: (A) autochory, when dispersal is carried out by the plant itself, and (B) allochory, when seeds are dispersed externally by abiotic and biotic factors.

The analysis of the two general dispersal systems provides insight into the intricate network of seed dispersal, the primary types of dispersal, and their derivatives (Figure 1).

#### 3.4.1 Dispersion in Autonomous Mode: Autochory

Autochory comprises self‐dispersal mechanisms (ballochory, blastochory, and herpochory) that actively eject or move diaspores a short distance from the parental plant (van der Pijl 1972; van Rheede van Oudtshoorn and van Rooyen 1999). Autochory exhibits several variants, including ballistic dispersal (Figure 1), which occurs when the diaspore is ejected forcefully because of explosive fruit dehiscence and whose dispersal is known as ballochory (Biswas and Bordolui 2021). This process is actuated by turgor tension in dead hygroscopic tissues or living tissues inside the fruit (van der Pijl 1982; Biswas and Bordolui 2021) and the dehydrated valves of the fruit coat mechanically propel the seeds in various directions (Hayashi et al. 2009). The potential energy necessary for the explosive dispersal of diaspores accumulates during fruit ripening. Consequently, the distance the diaspore travels from the parent plant depends on the total amount of energy released during dehiscence (van Rheede van Oudtshoorn and van Rooyen 1999). Autochory is effective for local spacing and avoiding immediate sibling competition in certain habitats (e.g., arid zones), but its limited range often requires secondary dispersal to achieve landscape‐scale recruitment (Vittoz and Engler 2007; Hailemariam 2021).

#### 3.4.2 Agent‐Dependent Dispersion: Allochory

In addition to autochory, most plant species employ allochory as a dispersal system, whereby abiotic or biotic factors act as external dispersion agents and encompass the majority of long‐distance events; it includes, besides wind and water as described above, animal vectors. They produce highly variable seed shadows depending on agent mobility and behavior (Correa et al. 2015; Vargas et al. 2023). These dispersal systems enhance the likelihood of diaspore dissemination beyond the maternal plant into novel, optimal environments adequate for germination and establishment (Chen et al. 2018).

Abiotic factors serve as primary agents of diaspore dispersal for numerous plant species (Sánchez‐Salas et al. 2017; Correa et al. 2015). Although specific morphological and physiological specializations are often present to facilitate effective dispersal through this environmental system (Biswas and Bordolui 2021), it is imperative to recognize that such specialized adaptations are not always strictly necessary for successful dispersal by a given abiotic mechanism. In contrast, biotic factors comprise the dispersal of diaspores by different animal groups. Biotic allochory often provides directed deposition into favorable microsites, whereas abiotic allochory yields more stochastic patterns that depend on environmental context (González‐Varo et al. 2015).

### 3.5 Controlled Dispersion by Biological Agents

A substantial proportion of seed‐bearing plants, estimated at approximately 75%, are reliant on various species of animals, including birds, mammals, ants, fish, and reptiles, to facilitate seed dispersal, a process termed zoochory (Figure 1), to facilitate the movement of seeds to microsites conducive to recruitment (Beckman and Sullivan 2023; Rogers et al. 2021; Sinu et al. 2020). The estimated number of plants with fleshy fruits dispersed by animals varies widely, with the main dispersal mechanism being endozoochory in up to 94% of woody plants, depending on the region (Jordano 2000; Buitrón‐Jurado and Ramírez 2014). In tropical ecosystems, frugivorous animals are responsible for the dispersal of fleshy diaspores in up to 90% of species and between 30% and 50% of temperate forest species (Aizen et al. 2002; Herrera 2002).

#### 3.5.1 The Role of Animals as Dispersers: The Zoochory

Zoochory is the dispersal of diaspores that are transported on the exterior (e.g., hair and feathers) or interior (e.g., digestive tract) of an animal's body (Iluz 2010). Animals can act as primary dispersers when the diaspores are removed directly from the parental plant or as secondary dispersers when already dispersed seeds are transported to more distant sites and thereby shape nonrandom spatial patterns of seed deposition (Howe and Smallwood 1982; Wang and Smith 2002).

The dispersal of diaspores by zoochory occurs through three distinct mechanisms (Gelmi‐Candusso et al. 2017). Seeds can be ingested, transported within the digestive tracts of animals, and expelled through excretion or regurgitation (endozoochory). Alternatively, seeds can be actively carried in hands, mouths, beaks, or jaws and stored or dropped after the edible part of the fruit has been removed (synzoochory). Finally, seeds can be passively carried while attached to the skin, fur, or feathers (epizoochory) (Howe and Smallwood 1982). The dispersal quality (legitimacy) and quantity (efficiency) of animal vectors depend on feeding behavior, gut passage effects, movement ecology, and defecation patterns (Nathan et al. 2008; Schupp et al. 2010). The following section will delineate the essential characteristics that facilitate the successful dispersion of each mechanism.

#### 3.5.2 Seed Transport by Ants: The Myrmecochory

The term “entomochory” refers to the dispersal of seeds, fruits, spores, and conidia by insects (Li Vigni and Melati 1999). Ants are, by far, the most prominent insects responsible for this form of dispersal, though other insects, such as hornets (vespicochory; Jules 1996), or dung beetles, which perform this function by transporting balls of excrement that may contain seeds. This process facilitates the mobilization of seeds, thereby promoting the establishment of seedlings from the seed bank in feces because of the beetles' activity (Ocampo‐Castillo and Andresen 2018). Other, smaller insects are attracted to the nutritive properties (elaiosomes) of seed‐bearing fruits and may contribute to dispersal in a similar manner (Gerola 1968). Evidence suggests that plants and dispersing insects have evolved in a strictly correlative manner, as the fruits of some plants may have originally evolved to attract dispersing insects (Li Vigni and Melati 1999).

In the context of insect dispersal, myrmecochory emerges as a notable phenomenon. Myrmecochory can be defined as the specific mode of seed dispersal facilitated by ants through the epizoochory system. In this system, ants have the function of transporting and storing diaspores, consuming rewards (e.g., arils), and leaving intact seeds in nutrient‐rich, predator‐safe microsites (Chapman and Onderdonk 1998; Iluz 2010). This mechanism is particularly important for herbaceous and understory species and frequently forms one phase of diplochory when coupled with ballistic or abiotic release (Chen et al. 2019; Ohtsuka et al. 2020).

#### 3.5.3 Seed Consumption and Seed Dispersal: The Endozoochory

Seed dispersal mechanisms encompass a diverse array of ecological interactions, among which endozoochory, dispersal via ingestion and defecation by animals, plays a central role. The frugivorous vertebrates represent the initial stage of primary dispersal for the propagules of numerous plants (Montiel and Montaña 2000). Endozoochory is a mutualistic relationship between plants and animals that has evolved concurrently. However, it is important to consider these plant–animal mutualistic interactions as an evolutionary “conflict of interests”, because both species try to maximize the benefit for themselves, independently of what happens to the other species (Fleming 1988). In this relationship, plants produce fleshy seed‐bearing fruits that are consumed by animals, and the seeds are later dispersed when the animals defecate or regurgitate (Cypher and Cypher 1999; Iluz 2010). This relationship is beneficial to the plant because gut passage can scarify hard coats and alter germination timing, but may also damage embryos, so effects are species‐and context‐dependent (Beckman and Sullivan 2023; Gardener et al. 1993; Traveset et al. 2001, 2007).

The effectiveness of endozoochory as a means of dispersing seeds and promoting germination and establishment in heterogeneous habitats situated at a distance from the source plant is well established and widely accepted (Giombini et al. 2024). Additional factors influencing the efficacy of long‐distance dispersal include the velocity of movement and the intestinal capacity of the animals, which enable the transportation of seeds over several kilometers, the dimensions of the animal species, its feeding habits, behavior, and its home range (Cousens et al. 2010). Similarly, internal anatomical factors, such as the pH of the stomach (which is associated with scarification) and the size of the digestive tract (which is associated with the size of the animal), are related to the retention time of the seeds and, consequently, to long‐distance dispersal (Rubalcava‐Castillo et al. 2023).

Although endozoochorous plant species often exhibit traits that attract frugivores, such as fresh fruits and nutritional rewards (Jordano 2000; Schupp et al. 2010), this mechanism is not restricted to taxa with classical frugivore‐associated syndromes. Empirical evidence shows that seeds from plants traditionally classified under other syndromes, including anemochory and thalassochory, can also be dispersed through endozoochory, particularly by generalist or opportunistic feeders (Karimi et al. 2020; Almeida et al. 2025). For instance, waterfowl have been documented dispersing seeds of aquatic and wind‐dispersed plants via gut passage, challenging the predictive power of morphological syndromes alone (Almeida et al. 2025). This functional overlap highlights the context‐dependent nature of dispersal processes and the need to integrate behavioral, physiological, and ecological dimensions when characterizing seed dispersal interactions (Yadav et al. 2025).

#### 3.5.4 A Variant of Endozoochory: “Diploendozoochory” (Analysis of the Effectiveness of Seeds on Digestion in Two Different Animal Species)

Despite the attributes of diaspores, there is mounting evidence that the animals that dislodge them from the maternal plant do not inevitably determine the fate of seeds. Alternatively, the seed dispersal process can involve multiple dispersal species, leading to complex interaction chains that influence the ultimate fate of the seeds. For instance, initial seed uptake by a primary disperser can be followed by subsequent handling by ants, dung beetles, or rodents, influencing a seed's journey from its initial consumption to its final deposition or destruction (Andresen and Urrea‐Galeano 2022; Ozinga et al. 2004).

**FIGURE 2:**
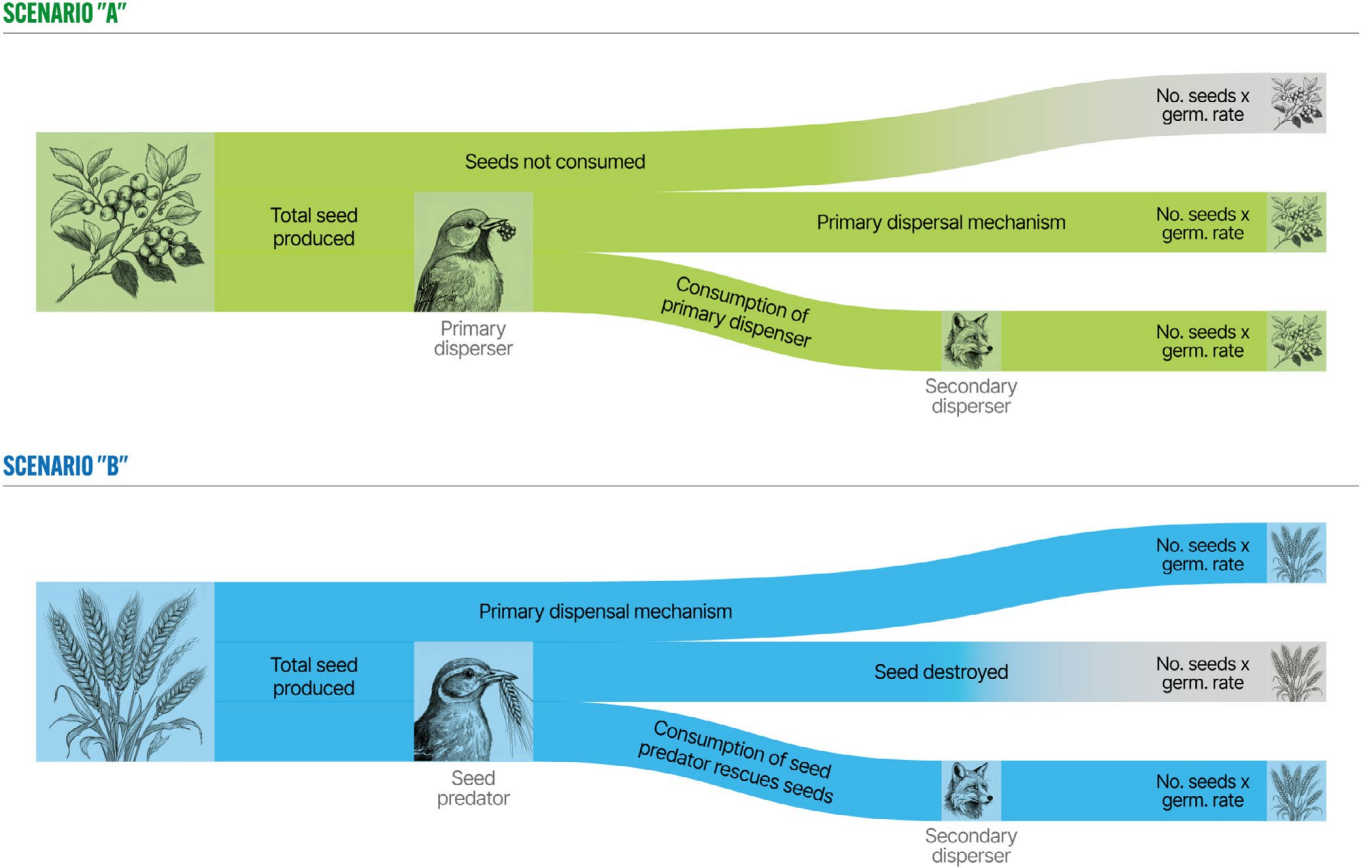
The seed dispersal process by diploendozoochory, in which seed‐bearing fruits are removed by a first disperser (prey) and may be expelled within scat before being hunted by a natural predator that could act as a secondary disperser. The illustration is based on the design of Hämäläinen et al. (2017).

This multiphase dispersal, often termed secondary dispersal, is crucial because different animal guilds can exert distinct pressures and provide varied benefits or detriments to seed survival and establishment (Vander Wall and Longland 2004). There is currently a growing interest, also known as “diplochory” or “indirect dispersal”. However, relatively little attention has been devoted to the phenomenon of “diploendozoochory,” which encompasses the dispersal of seeds by multiple species of animals, frequently involving sequential gut passage through two animal species (commonly prey and predator), a multiphase chain that can extend dispersal distance and modify germination via cumulative digestive effects (Hämäläinen et al. 2017; Nogales et al. 2007) (Figure 2).

Secondary dispersal is a legitimate process, given that when carried out by wide‐ranging carnivores, it allows for the colonization of habitats disturbed by climate change (Nogales et al. 2012), including remote islands, or it can locally influence the number of seeds entering an area.

A key aspect of secondary dispersal is understanding how the germination potential of seeds can change after passing through the digestive tract of carnivores, when these animals are considered legitimate dispersers. Double digestion may increase germination percentage in some cases because of a longer intestinal retention time (Nogales et al. 2015); of some carnivores, the number of seeds dispersed by them is generally low. Ultimately, the number of seeds dispersed by carnivores depends on the number of seeds consumed by the primary disperser (Rubalcava‐Castillo et al. 2023). Evidence suggests that diploendozoochory can be ecologically relevant when seeds are deposited in different nutrient or microhabitat contexts (Hämäläinen et al. 2017; Sarasola et al. 2016).

#### 3.5.5 Triploendozoochory: A Special Case Study

The role of predators in secondary seed dispersal is gaining attention and inspiring new research. Building on the established concept of diploendozoochory, we now propose a testable hypothesis: triploendozoochory. This is the sequential passage of seeds through three vertebrates linked in a food chain (e.g., herbivore to mesopredator to top predator). In food nets with a primary consumer followed by two predators, seeds may undergo three rounds of endozoochorous dispersal. This process could greatly influence seed distribution and ecosystem dynamics.

**FIGURE 3:**
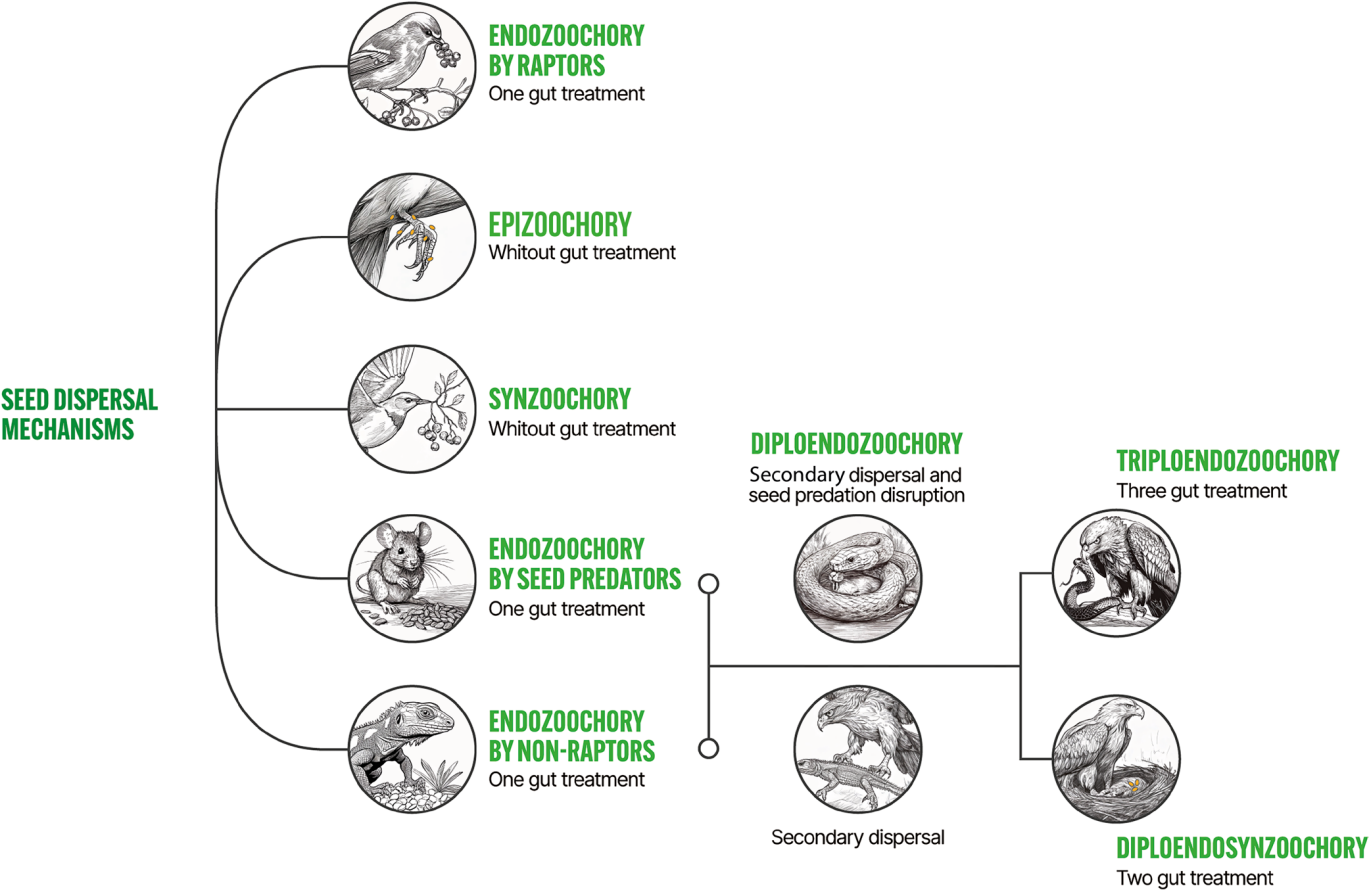
Main mechanisms of dispersal by zoochory. The endozoochory illustrates the representation of the possible triploendozoochory process in a food chain formed by: (1) mouse (prey), (2) snake (predator), and (3) eagle (top predator).

This concept of triploendozoochory, although speculative, offers a compelling avenue for exploring the complexities of seed dispersal and its ecological implications. For instance, this phenomenon can be observed in the sequence of a mouse, a snake, and a bird of prey (Figure 3). In this sense, there is evidence of diploendozoochory of raptors (Pérez‐Méndez and Rodríguez 2018) and snakes, the latter when preying on mice (Reiserer et al. 2018; Schuett et al. 2022). In this trophic net, a triple endozoochory would occur if the mouse is predated by the snake and these by a bird of prey. Consequently, the seed that initially passed through the mouse tract could ultimately reach the bird of prey (Figure 3). Other food nets in which triploendozoochory can occur include those in which raptors prey on other frugivorous animals, such as lizards (Nogales et al. 2007).

This type of dispersal is complex. Triploendozoochory is therefore presented as a testable extension of diploendozoochory that may be important in complex food webs and islands or fragmented systems where predators move wider than the other species. New methodological approaches must be proposed to describe the functional importance of triploendozoochory in terms of legitimacy, efficiency, and effectiveness in seed dispersal and post‐dispersal, as well as in ecosystem conservation.

#### 3.5.6 Carrying Seeds? Dispersal Carried Out Over the Outside of Animals: The Epizoochory

Epizoochory is another variant of zoochory; it represents a type of seed dispersal carried out on the external body surfaces (fur, feathers, mucous membranes). This mechanism has significant potential for dispersal over intermediate to long distances and may play an important role in the dynamics of metapopulations concerning the spatial distribution of seeds (Higgins et al. 2003; Adriaens et al. 2007).

Epizoochoric dispersal comprises two essential stages. First, the seed must contact and attach to an animal (seed attachment) that will act as the dispersal vector, which determines the number of seeds dispersed by the animal's coat. Second, the seed must be retained long enough for dispersal to occur; that is, the shedding of seeds, which determines where the seeds are deposited (Will et al. 2007; Sato et al. 2023). For this process to occur, transported plants have evolved various external specializations conducive to this type of dispersal. These specializations include the production of adhesive mucus, which enables the plant to adhere to animal body parts when mixed with mud (Figuerola and Green 2002). Additionally, a range of hooks, barbs, thorns, and spikes are present on the diaspora, which are also utilized against predators (Iluz 2010).

The success of long‐distance dispersal via epizoochory critically depends on the effective, often haphazard, plant phenology, the stochastic adhesion of seed‐bearing fruits to animal body surfaces, and the animal's body height. The latter influences the duration and distance of seed transport by unaware species (Sorensen 1986; Sato et al. 2023). Although less quantified, endozoochory can achieve substantial distance when large‐bodied, mobile animals are involved and can act as complementary dispersal to endozoochory (Hernández‐Brito et al. 2021; Sato et al. 2023).

#### 3.5.7 What Do They Keep in There? The Dispersion of Storage: The Synzoochory

Synzoochory is a form of diaspore dispersal characterized by animals collecting and catching seeds (hoarding), typically for future consumption. Consequently, the animal participating in this interaction plays a dual role, acting both as a seed disperser and a seed predator (Gómez et al. 2019). The term synzoochory was first introduced by Dixon (1933) and is derived from the Greek prefix “syn,” meaning “together,” which refers to the action of moving the seed in conjunction with the animal. Synzoochory entails the intentional movement and storage of seeds by animals, although this process may result in the loss of some seeds (Gómez et al. 2019). In some cases, stored seeds may not be consumed because of the animal's inability to retrieve them, either from forgetfulness or misplacement (Vander Wall 2001). Animals are directly attracted to seeds as a primary resource, often for extracting the endosperm or embryo (Hulme 2002). This direct attraction contrasts with dispersal mechanisms where animals are drawn to seeds by secondary fruit traits such as the appearance, odor, or fleshiness (Gómez et al. 2019). Therefore, synzoochory does not involve ingestion and scarification processes through the digestive tract, nor does it depend on the presence of adhesive seeds (Osorio‐Zuñiga et al. 2014). The net effect depends on caching behavior, recovery rates, and cache locations, and it frequently functions as one phase within polychory or diplochory chains.

#### 3.5.8 The Combination of Dispersion Systems (Polychory/Diplochory)

Most natural seed dispersal is typically a complex process that occurs in multiple phases: initial release is often followed by secondary or tertiary movements (e.g., 1. Barochory, 2. Synzoochory, 3. Endozoochory). Following the maturation of the diaspores, they are removed directly from the mother plant by gravitational forces, wind, or various animals (e.g., birds, mammals, and reptiles). In the initial phase, the seed‐bearing fruits may be dispersed in a preliminary movement away from the maternal plant. The primary dispersal system is the simplest; however, some complex forms of seed dispersal may comprise two or more phases (secondary dispersal), referred to as diplochory and polychory (e.g., repeated storage of a nut by the same or different animals). Thus, the term diplochory refers to the dispersal of seeds in two separate phases, usually associated with different dispersal structures and exhibiting different types of specializations to different dispersal agents (Vander Wall and Longland 2004). These different forms of dispersal have varying effects on the final fate and viability of the seeds. It is widely acknowledged that the combination of multiple dispersal mechanisms often confers greater reproductive benefits than reliance on isolated means (Czarnecka and Kitowski 2013). However, it is equally important to recognize that certain combinations of dispersal systems can incur negative consequences. For instance, an enhanced process of seed dispersal through one mechanism might inadvertently lead to an increase in seed predation (Gong et al. 2015) or undesirable post‐dispersal density effects (Wang 2020), ultimately diminishing overall recruitment success. This highlights the complex interplay between dispersal strategies and subsequent ecological filters.

Secondary dispersal represents a phenomenon that not only facilitates an increase in dispersal distance but also reduces the probability of seed predation because of density dependence (Vander Wall and Longland 2004). Diplochory is gaining recognition as a common means of seed dispersal in temperate and tropical ecosystems, because a significant portion of seeds has been shown to be able to disperse through multiple systems rather than just one (Vander Wall and Longland 2004; Correa et al. 2015). This has the beneficial effect of ensuring the reproduction of plants and thus the continued survival of the species (Vander Wall and Longland 2004).

To synthesize the diversity of dispersal mechanisms described above, Table 2 summarizes key traits, conditions, and ecological roles across all modes discussed in Section 3.

**TABLE 2:** Summary of dispersal modes.

Dispersal mode	Mechanism (2–3 words)	Key conditions	Typical timescale/distance	Ecological relevance
Barochory	Gravity/drop	Largue, heavy diaspores; low appendages	Seconds → meters (short)	Local recruitment; high seed density; often precursor to secondary dispersal
Anemochory	Wind lofting (wings, pappus)	Updrafts, low mass, surface roughness	Seconds‐hours → short to long (depends on updraft)	Long‐distance potential; sensitive to weather and habitat structure
Hydrochory	Float/current transport	Buoyant tissues, impermeable coatings	Hours‐days → local to long (currents)	Colonization of waterways/coast; shell/coat traits crucial
Autochory	Self‐ejection (ballistic, hygroscopic)	Fruit mechanics, small‐scale energy release	Seconds‐minutes → meters	Shot‐range spacing; limits gene flow without secondary vectors
Allochory	External agents (abiotic/biotic)	Depends on vector (wind, water, animals)	Variable (sec → years)	Majority of long‐distance events; directed vs. random deposition
Zoochory	Animal‐mediated (general)	Frugivory, movement, gut/behavior traits	Hours‐days → often long	Directed deposition, seed treatment (scarification), and high ecological impact
Myrmecochory	Ants bury seeds (elaiosomes)	Elaiosome presence; ant behavior	Minutes → months (caching)	Safe, nutrient‐rich microsites; important for understory/herbs
Endozoochory	Ingestion defecation/regurgitation	Frugivory, gut retention, and digestive chemistry	Hours‐days → potentially long	Scarification or damage; directed deposition (perches, trails)
Diploendozoochory	Sequential gut passage (prey, predator)	Predation chains; seed survival through two guts	Days → extended distances	Can extend distances/change deposition context; variable effectiveness
Triploendozoochory	Three sequential gut passages in trophic chains	Multi‐trophic predation, seed survival through three guts	Days‐weeks → potentially very long	Hypothetized rare long‐distance events; testable multiphase route
Epizoochory	External attachment (fur, feathers mucilage)	Hooks, mucilage, long fur/feathers	Minutes‐days → intermediate to long	Effective when attached to wide‐ranging animals; Complementary to endozoochory
Synzoochory	Caching/hoarding (intentional)	Hoarding species (rodents, corvids)	Days → years (cached)	Dual role: dispersal and predation; forgotten caches enable recruitment
Polychory	Sequential modes (e.g., baro‐syn‐endo)	Overlap of vectors & phases	Composite of component timescales	Alters kernels and seed condition; central to realistic dispersal models

## 4 Dispersal Multi‐Phase

This manuscript's main contribution is a conceptual re‐evaluation of seed dispersal as an inherently multiphase process: rather than treating dispersal modes as isolated categories, we argue that sequential and simultaneous phase transitions (abiotic ↔ biotic) are central to understanding dispersal effectiveness, gene flow, and ecosystem resilience. This perspective reduces the catalog‐like tone by prioritizing functional routes, interaction chains, and the filters that act at each phase, and by highlighting research and conservation implications.

### 4.1 Conceptual Framing

Seed dispersal is increasingly recognized not as a single event but as a dynamic, multiphase process shaped by sequential overlapping interactions among biotic and abiotic agents. This expanded view reframes dispersal as a network of transitions, each with distinct filters, risks, and ecological consequences, rather than a linear trajectory. Multiphase dispersal is especially relevant under global change, where fragmentation, altered vector communities, and shifting phenologies disrupt traditional dispersal routes and favor novel combinations (Schupp et al. 2010; Travis et al. 2013).

### 4.2 Definitions and Phase Properties

1. Phase definition: We defined three operational dispersal phases: primary release (detachment from parent), secondary transit (re‐handling, gut passage, attachment), and deposition/post‐dispersal fate (cache, gut egesta, substrate incorporation). Each phase imposes different abiotic and biotic filters that alter seed condition, probability of survival, and the special outcome (Culot et al. 2015; Traveset et al. 2014; Vander Wall and Longland 2004) (Table 3).
2. Interaction of abiotic and biotic factors. Abiotic forces (wind, water, and gravity) and biotic agents (frugivores, carnivores, herbivores, and ants) often act sequentially or concurrently: for example, a pappus bearing seed may loft by wind (primary), land on a trail and be ingested by a granivore (secondary), then be consumed by a predator whose scat deposits the seeds elsewhere (tertiary). Each transition modifies dispersal kernel, seed coat integrity, dormancy breaking, and deposition microhabitat (Godó et al. 2023; Nathan et al. 2008; Navarro‐Ramos et al. 2024).
3. Consideration must be given by timescales and transit durations. Transit durations are known to vary according to vector and phase, and they have been shown to strongly influence distance. Wind events act within the range of seconds to hours, frugivore gut retention ranges from hours to days, predator‐mediated secondary retention may add many hours, and caches can delay germination for months or even years. These temporal parameters interact with the field of vector movement ecology to establish plausible dispersal distances (Figuerola and Green 2002; Rubalcava‐Castillo et al. 2023).
4. The consequences of survival and establishment must be considered. The sequential phases of the germination process have been shown to create compound filters, whereby the presence of the gut passage can either enhance or reduce the viability of the seeds. For example, scarification can enhance germination, whereas embryo damage can reduce viability. Additionally, caching has been observed to reduce predator risk but may also place seed in suboptimal microhabitats. Furthermore, predator mediated phases have been demonstrated to increase long‐distance colonization, but to reduce the number of seeds dispersed. The quantification of the trade‐off between distance and per‐seed recruitment probability is central to the evaluation of dispersal effectiveness in multiphase systems (Hämäläinen et al. 2017; Schupp et al. 2010).
5. Research priorities and methodological needs. We recommend integrated approaches combining movement data (GPS/telemetry), gut retention experiments, seed viability assays, and spatial models to estimate multiphase dispersal kernels. Standardized reporting of transit durations, seed condition metrics, and deposition microhabitats will enable comparison across systems and improve predictive models of colonization and gene flow (Culot et al. 2015; Nathan et al. 2008).

**TABLE 3:** Operational phases of seed dispersal, detailing mechanisms, timeframes, changes in seed status, and ecological consequences.

Phase	Definition	Typical mechanisms	Typical timescale	Main seed condition change	Principal ecological consequence
Primary release	Detachment from the parent plant	Ballistic, gravity, wind, and first ingestion	Seconds‐hours	Intact or initial abrasion	Sets initial seed density and local kernel
Secondary transit	Re‐handling, vector switches	Gut passage, caching, attachment, water re‐entry	Minutes‐days‐months	Scarification, embryo damage, buoyancy loss/gain	Alters germination probability and dispersal distance
Deposition/post‐dispersal fate	Final placement and early fate	Defecation, regurgitation, cache abandonment burial	Hours‐years	Burial, incorporation into a seed bank, predation	Determines recruitment success and local establishment

### 4.3 Mechanistic Transitions and Filters

Each phase in a multiphase dispersal chain imposes distinct filters: physical (abrasion and flotation), chemical (digestive enzymes and pH), biological (predation and microbial colonization), and spatial (vector movement and habitat boundaries). For example, a seed digested by a frugivore may be scarified, enhancing germination, but if later consumed by a predator (diploendozoochory), cumulative digestive exposure may reduce viability or delay germination (Nogales et al. 2007; Rubalcava‐Castillo et al. 2023). Similarly, seeds attached externally (epizoochory) may be dislodged during grooming, enter endozoochory if ingested during preening, or be eaten by a predator as part of the prey, creating unexpected transitions (Will et al. 2007).

Temporal dynamics also vary: some transitions occur within minutes (e.g., ballistic released → ant collection), whereas others span months (e.g., caching → overwintering → flooding → germination). These timescales interact with vector movement ecology to determine effective dispersal kernels and colonization potential (Figuerola and Green 2002; Nathan et al. 2008).

### 4.4 Multiphase Dynamics Across Ecosystems

In aquatic systems, waterbirds act as key multiphase vectors by combining ingestion (endozoochory), external transport (epizoochory), and cross‐boundary movement between aquatic and terrestrial habitats. Seeds of aquatic plants such as Potamogeton and Myriophyllum are ingested by ducks and later defecated into terrestrial wetlands, or vice versa, enabling bidirectional dispersal across habitat types (Green et al. 2023). These transitions often involve changes in seed buoyancy, microhabitat colonization, and exposure to digestive enzymes, which alter germination probability and dispersal distances.

In arid environments, multiphase dispersal frequently involves autochory followed by myrmecochory or synzoochory. For example, desert legumes may explosively release seeds (ballochory), which are then collected by ants and buried in shaded microsites, or hoarded by rodents in burrows (Vander Wall and Longland 2004). These transitions buffer seeds against extreme temperatures and predation, and may delay germination until favorable conditions arise (Chen et al. 2019).

In tropical forests, fruit bats (chiropterochory) play a central role in nocturnal dispersal chains. Bats ingest fruits and defecate seeds during flight, often over long distances, but also drop partially eaten fruits that are later consumed by terrestrial frugivores or decomposers (Fujita and Tuttle 1991; Kelm et al. 2008). This creates a cascade of interactions where seeds may experience multiple gut passages, microbiotal colonization, and deposition in diverse microhabitats. Chiropterochory is particularly important for early successional and canopy species and complements bird‐mediated dispersal during night–day phases (Muscarella and Fleming 2007).

Ichthyochory, seed dispersal by fish, is an emerging multiphase mechanism in riparian and swamp ecosystems. Cyprinid fishes ingest fruits of riparian trees during seasonal floods and later excrete seeds downstream, often into oxbow lakes or floodplain forests (Horn et al. 2011). These seeds may then be consumed by waterbirds or deposited in sediments, initiating further dispersal phases. Although undocumented, ichthyochory may be critical for connectivity in tropical river systems (Correa et al. 2015).

Microbial dispersal via animal guts, especially by waterbirds, is another underexplored multiphase pathway. Resting eggs of aquatic invertebrates and microbial propagules can survive gut passage and be deposited in distant wetlands, often with altered microbial communities due to gut flora interchange (Green et al. 2023). These interactions suggest that dispersal multiphase extends beyond seeds to include entire propagule networks, with implications for biogeochemistry and metacommunity dynamics (Table 4).

**TABLE 4:** Representative examples of multiphase dispersal in ecosystems, emphasizing the diversity of vectors and transitions involved.

Ecosystem	Example taxa	Phases involved	Key transitions	Ecological outcome
Aquatic	*Potamogeton*, *Myriophyllum*	(1) Endo, (2) Epizoo, (3) Hydro	(1) Duck ingestion, (2) feather attachment, (3) water deposition	Cross‐boundary dispersal; altered buoyancy
Arid	*Acacia*, *Prosopis*	(1) Auto, (2) Myrmeco, (3) Synzoo	(1) Ballistic release, (2) ant burial, (3) rodent caching	Delayed germination; predator‐safe microsites
Tropical forest	*Ficus*, *Cecropia*	(1) Endo, (2) Drop, (3) Endo	(1) Bat ingestion, (2) fruit drop, (3) terrestrial frugivore ingestion	Extended dispersal; diverse microhabitats
Riparian	*Barringtonia*, *Ficus*	(1) Endo, (2) Hydro, (3) Endo	(1) Fish ingestion, (2) water transport, (3) bird ingestion	Long‐distance dispersal; aquatic‐dispersal link
Wetland microbial	*Rotifers*, *algae*	(1) Endo, (2) Epizoo, (3) Hydro	(1) Waterbird ingestion, (2) feather transport, (3) wetland deposition	Microbial colonization; metacommunity expansion

Table 5 presents representative multiphase dispersal routes, including example taxa and expected outcomes for seed viability and spatial distribution.

**TABLE 5:** Representative multiphase dispersal routes, including examples of taxa and expected results in terms of seed viability and spatial distribution.

Route code	Typical sequence	Example vectors	Typical outcome for distance and viability
A	(1) Anemo, (2) epizoo, (3) endozoo	(1) Wind, (2) landing on fur, (3) ingestion by a grooming bird	Intermediate distance: viability depends on cumulative abrasion
B	(1) Baro, (2) synzoo, (3) endo (diploendo)	(1) Fruit drop, (2) rodent caching, (3) predator ingestion	Extended distance: fewer seeds but deposited in different microhabitats
C	(1) Endo, (2) hydro	(1) Frugivore ingestion, (2) defecation into water	Changed buoyancy: often reduced hydrochorous capacity post‐gut passage
D	(1) Auto, (2) myrmeco	(1) Ballistic ejection, (2) ant collection	Local redistribution to nutrient‐rich microsites; high seed survival
E	(1) Synzoo, (2) epizoo	(1) Caching by a rodent, (2) external attachment to a passing mammal	Potential unexpected long‐distance jump; low frequency but high impact

### 4.5 Conceptual Synthesis and Modeling Needs

To operationalize multiphase dispersal, we propose a network‐based framework where nodes represent dispersal phases and edges represent transitions. Each edge can be weighted by probability, duration, and ecological outcome (e.g., viability and distance). This approach allows integration of empirical data (e.g., gut retention and seed survival) with spatial models and can accommodate stochastic and context dependence (Green et al. 2023; Travis et al. 2013). Network metrics (e.g., centrality and modularity) may reveal key vectors or bottlenecks in dispersal systems, guiding conservation and restoration strategies.

Methodologically, multiphase dispersal requires interdisciplinary tools: stable isotope tracing. DNA barcoding of seeds in scats, telemetry of vector movement, and experimental feeding trials. Combining these approaches can reveal hidden dispersal routes and quantify their contribution to recruitment and gene flow (Horn et al. 2011; Reiserer et al. 2018).

Finally, recognizing multiphase dispersal has implications for conservation: protecting large‐bodied frugivores and predators may sustain long‐distance dispersal chains, while restoring wetlands and riparian corridors may reactivate aquatic‐terrestrial transitions. In fragmented landscapes, artificial corridors or assisted dispersal may mimic lost phases and restore connectivity (Green et al. 2023; Schupp et al. 2010).

## 5 Triploendozoochory and Its Role in Dispersal Multiphase

We defined triploendozoochory as the sequential passage of a diaspore through three distinct animal digestive systems along a trophic net, each passage representing an endozoochorous phase that cumulatively modifies seed position, condition, and deposition context. Triploendozoochory is proposed here as a testable instance of multiphase dispersal rather than as a widespread, fully documented phenomenon (Rubalcava‐Castillo et al. 2023).

### 5.1 Evidence Base and Literature Context

Empirical support for sequential multi‐predator chains is limited but growing: diploendozoochory has been demonstrated in mammals and birds (Godó et al. 2023; Nogales et al. 2007; Sarasola et al. 2016), and captive and field studies indicate that cumulative digestion can alter germination probability (Nogales et al. 2015; Rubalcava‐Castillo et al. 2023). Triploendozoochory remains underrepresented in the JCR literature and requires targeted observation and experimental designs; we therefore include this concept as a research hypothesis and invite focused tests (Clifford and Monteith 1989) to simulated empirical validation.

### 5.2 Proposed Steps and Mechanistic Trajectory

1. Stepwise sequence: (i) primary ingestion by a herbivore/omnivore, producing initial gut passage effects and patch‐level movement; (ii) predation on that primary consumer by an intermediate predator that ingests the seeds present in prey gut or body; (iii) subsequent predation on the intermediate predator by an apex predator whose larger home range and defecation/rub behavior deposit seeds at broader spatial scales (Table 6). Each step must be empirically tracked via diet analysis (scat/pellet), seed recovery, viability testing, and movement ecology of vectors (Padilla and Nogales 2009; Reiserer et al. 2018).
2. Conditions conducive to triploendozoochory. The necessary conditions for the occurrence of such chains include frequent predation across trophic levels, sufficient seed survival after multiple digestive exposures, and predators with large or directed ranges. Island ecosystems, fragmented landscapes, and predator‐rich food nets may be hotspots for such chains (Nogales et al. 2012; Sarasola et al. 2016).
3. Relative importance and knowledge gaps. Triploendozoochory likely contributes to rare long‐distance colonization events and alters seed fate in ways not captured by single‐phase studies. However, its relative contribution to population‐level recruitment is unknown and probably low per event; nonetheless, rare events can have outsized biogeographic consequences (Higgins et al. 2003; Nathan et al. 2008). Priorities include controlled feeding trials, molecular tracing of seed provenance in predator scats/pellets, and models linking trophic movement networks to dispersal kernels.

**TABLE 6:** Proposed steps for triploendozoochory, biological requirements for each phase, and empirical signals needed to detect them in the field or in experiments.

Step	Biological process	Necessary conditions	Empirical signals to detect
1	Primary consumption by herbivore/omnivore	Prey consumes seeds; seeds survive the initial gut	Seeds recovered intact from prey scats/direct observation
2	Predation on the primary consumer by the mesopredator	Frequent prey capture, seed survival after predator digestion	Seeds found in mesopredator scats/pellets with identifiable prey remains
3	Predation on mesopredator by apex predator	Apex predator ingests mesopredator; large‐ranging behavior	Seeds recovered in apex predator scats/pellets; telemetry shows wide movement
Overall	Cumulative effect	Seed tolerance to repeated digestion; overlapping trophic links	Molecular/stable‐isotope tracing linking seed origin to successive scats; controlled feeding trials

In addition to triploendozoochory, recent literature has proposed diploendosynzoochory as a hybrid dispersal mechanism that combines sequential ingestion and predator‐mediated transfer (diploendozoochory) with post‐predation caching or manipulation by a third agent (synzoochory) (Figure 3). This route may occur when carnivores fed on frugivorous prey containing viable seeds and subsequently deposit scats that are later handled or hoarded by rodents, beetles, or birds (Hämäläinen et al. 2017; Pérez‐Méndez and Rodríguez 2018; Vander Wall and Longland 2004).

Although empirical documentation remains limited, diploendosynzoochory represents a plausible multiphase pathway in fragmented or predator‐rich ecosystems, where carnivore scats attract secondary dispersers. These interactions may enhance seed burial, reduce predation risk, and increase microsite heterogeneity, ultimately influencing recruitment success (Godó et al. 2023; Pérez‐Méndez and Rodríguez 2018).

We recommended future studies to explore this mechanism using combined diet analysis, camera traps, and seed fate tracking, specifically in systems where carnivore scats are known to attract caching species. Diploendosynzoochory may be particularly relevant in dry forests, scrublands, and montane habitats where trophic overlap and scavenging behavior are common (Hämäläinen et al. 2017).

### 5.3 Research Recommendations and Conservation Implications

Testing triploendozoochory will require integrative methods (stable isotopes/DNA for seed origin, captive trials for cumulative gut effects, and telemetry for predator movement) and could reveal underappreciated roles of predators in plant connectivity and restoration, especially where large carnivores have been extirpated or reintroduced (Sarasola et al. 2016). Recognizing triploendozoochory as a possible multiphase route reframes predator conservation as also contributing to plant dispersal services and landscape resilience.

## 6 Conclusions

1. Seed dispersal is a dynamic and complex process that can occur through various mechanisms. This complexity arises from evolutionary processes (genetic variation) whereby plants have developed specific specializations in response to their environments that facilitate the avoidance of competition among closely related descendants. Nevertheless, it is reasonable to posit that seed size may act as a limiting factor when dispersed by the plant itself (Moles et al. 2005, 2007). Consequently, secondary dispersal, which may occur via endozoochory or hydrochory, among other mechanisms, can hold significant ecological value by increasing dispersal distances.
2. Furthermore, ecological interactions contribute to the complexity of seed dispersal, as relationships with other organisms, such as prey and predators, along with habitat characteristics, influence the efficiency of the process. For this reason, we propose the possibility of a close relationship within trophic networks where multiple predators could act as dispersers, resulting in a phenomenon we term triploendozoochory. Although rare and undocumented, the sequential passage of diaspores through three tropically linked vertebrates offers a compelling example of how predator–prey dynamics can shape plant connectivity.
3. Multiphase dispersal emerges as a unifying paradigm that captures the ecological realism of seed movement in heterogeneous landscapes. We conclude that future research should prioritize the quantification of multiphase dispersal routes, the identification of key vectors and transitions, and the integration of movement ecology, gut passage experiments, and molecular tracing. Recognizing the multiphase nature of seed dispersal not only advances ecological theory but also informs conservation strategies aimed at restoring functional connectivity and resilience in changing landscapes.

## Author Contributions

**Fabián Alejandro Rubalcava‐Castillo:** conceptualization (equal), data curation (lead), formal analysis (lead), funding acquisition (equal), investigation (lead), methodology (lead), project administration (equal), resources (equal), software (equal), supervision (lead), validation (equal), visualization (lead), writing – original draft (lead), writing – review and editing (lead). **Martha Susana Zuloaga‐Aguilar:** conceptualization (equal), data curation (lead), formal analysis (equal), investigation (equal), project administration (lead), resources (equal), supervision (equal), validation (lead), visualization (equal), writing – original draft (lead), writing – review and editing (lead). **Luis Ignacio Íñiguez‐Dávalos:** conceptualization (equal), data curation (equal), investigation (equal), project administration (lead), supervision (equal), validation (equal), writing – original draft (lead), writing – review and editing (lead). **Víctor Manuel Martínez‐Calderón:** conceptualization (equal), data curation (equal), investigation (equal), supervision (equal), validation (equal), writing – original draft (equal). **Joaquín Sosa‐Ramírez:** conceptualization (equal), funding acquisition (equal), investigation (equal), resources (equal), supervision (equal), validation (equal), writing – original draft (equal), writing – review and editing (equal).

## Conflicts of Interest

The authors declare no conflicts of interest.

## Data Availability

This manuscript is a review article and therefore does not contain a Data Availability Statement, since there is no information from any experiment or field work.
